# Supporting self-management in women with pre-existing diabetes in pregnancy: a mixed-methods sequential comparative case study

**DOI:** 10.1186/s12912-023-01659-1

**Published:** 2024-01-02

**Authors:** Katelyn Sushko, Patricia Strachan, Michelle Butt, Kara Nerenberg, Diana Sherifali

**Affiliations:** 1 Faculty of Health Sciences, School of Nursing, McMaster University, 1280 Main Street West, Hamilton, ON L8S 4K1 Canada; 2 Departments of Medicine and Obstetrics & Gynecology, University of Calgary, Calgary, AB Canada

**Keywords:** Type 1 Diabetes, Type 2 Diabetes, Diabetes in pregnancy, Self-management, Qualitative research, Quantitative research, Mixed methods

## Abstract

**Introduction:**

Maternal glycemia is associated with pregnancy outcomes. Thus, supporting the self-management experiences and preferences of pregnant women with type 1 and type 2 diabetes is crucial to optimize glucose control and perinatal outcomes.

**Research design and methods:**

This paper describes the mixed methods integration of a sequential comparative case study. The objectives are threefold, as we integrated the quantitative and qualitative data within the overall mixed methods design: (1) to determine the predictors of glycemic control during pregnancy; (2) to understand the experience and diabetes self-management support needs during pregnancy among women with pre-existing diabetes; (3) to assess how self-management and support experiences helpe to explain glycemic control among women with pre-existing diabetes in pregnancy. The purpose of the mixing was to integrate the quantitative and qualitative data to develop rich descriptive cases of how diabetes self-management and support experiences and preferences in women with type 1 and type 2 diabetes during pregnancy help explain glucose control. A narrative approach was used to weave together the statistics and themes and the quantitative results were integrated visually alongside the qualitative themes to display the data integration.

**Results:**

The quantitative results found that women achieved “at target” glucose control (mean A1C of the cohort by the third visit: 6.36% [95% Confidence Interval 6.11%, 6.60%]). The qualitative findings revealed that feelings of fear resulted in an isolating and mentally exhausting pregnancy. The quantitative data also indicated that women reported high levels of self-efficacy that increased throughout pregnancy. Qualitative data revealed that women who had worked hard to optimize glycemia during pregnancy were confident in their self-management. However, they lacked support from their healthcare team, particularly around self-management of diabetes during labour and delivery.

**Conclusions:**

The achievement of optimal glycemia during pregnancy was motivated by fear of pregnancy complications and came at a cost to women’s mental health. Mental health support, allowing women autonomy, and the provision of peer support may improve the experience of diabetes self-management during pregnancy. Future work should focus on developing, evaluating and implementing interventions that support these preferences.

**Supplementary Information:**

The online version contains supplementary material available at 10.1186/s12912-023-01659-1.

## What is already known on this topic

• Pregnant women living with type 1 and type 2 diabetes have an increased risk of perinatal complications, including fetal and infant death.

• As maternal glycemia is associated with pregnancy outcomes, supporting women in diabetes self-management may optimize glycemia and reduce perinatal complications.

## What this study adds

• Women who achieved optimal glycemia during pregnancy reported high levels of self-efficacy in diabetes self-management.

• Diabetes self-management negatively impacted women’s mental health and made for an isolating pregnancy experience.

• Mental health support, peer support and autonomy in diabetes self-management is preferred by patients to improve their pregnancy experiences.

## How this study might affect research, practice or policy

• Peer support and mental health interventions were unavailable for study participants.

• Policies supporting maternal self-management of diabetes during labour and delivery were also lacking.

• Appropriate peer and mental health interventions, as well as policies to support autonomy of self-management during labour and delivery, are required.

• Future research should focus on developing interventions related to these desired supports and implementing them into the standard of care for this population.

## Introduction

With the rising prevalence of overweight and obesity and an older average maternal age during childbirth, type 2 diabetes in pregnancy has been steadily increasing [1–3]. The incidence of type 1 diabetes has also been rising, with an etiology that remains largely unknown [4]. These factors have contributed to the increased prevalence of pre-existing type 1 and type 2 diabetes in pregnancy, affecting 0.5–2.4% of pregnancies worldwide [5–9].

Pregnancies impacted by pre-existing diabetes are at an increased risk for many complications, from congenital anomalies to fetal and infant death [10]. Maternal glycemia, measured by glycosylated hemoglibin A1C (A1C), is closely linked to perinatal morbidity and mortality; each 0.1% increase of periconception A1C above 4.9% confers a 2% and 3% relative increase in fetal and infant death, respectively [11]. As a result, women experience a heavy burden of diabetes self-management during pregnancy, typically occurring outside of the health care system. During pregnancy, when additional stressors compound the stresses of everyday life, there may be an increased occurrence of mental health disorders. The prevalence of mental health disorders among adults with diabetes is already higher when compared to those without diabetes. Thus, women with diabetes in pregnancy may be even more likely to be affected by mental health disorders during pregnancy [12].

Supporting women in diabetes self-management is important to reduce mental stress, optimize glycemia during pregnancy and subsequently improve perinatal outcomes. How to best support women with pre-existing diabetes during pregnancy in self-management to attain optimal glycemia is not well understood. Currently, women with diabetes in pregnancy are supported in multiple ways [1]. Interventions include preconception care and counselling, care by a multidisciplinary team and education regarding the importance of self-monitoring of blood glucose, recommendations for weight gain and insulin administration, among others [1]. Throughout the duration of pregnancy, women attend appointments with the healthcare team to reinforce these concepts, including from endocrinologists and obstetricians to nurses and dietitians [1]. An exploration of this topic is therefore of importance to a variety of professionals of the multidisciplinary team.Women with pre-existing diabetes in pregnancy are unique in their self-management experiences and preferences compared to women with gestational diabetes. First, the management of pregnant women with pre-existing type 1 and type 2 diabetes is more complex than women with gestational diabetes due to their higher risk of experiencing serious perinatal complications and the need for insulin therapy [10]. Furthermore, in type 1 and type 2 diabetes, attention during pregnancy is focused on titrating insulin dosing using pens, continuous infusion sets (e.g., pumps) and continuous glucose monitors, while avoiding hypoglycemia. This is in contrast to the general focus on nutrition and exercise-related interventions for many women with gestational diabetes [1]. Glycemic targets during pregnancy among women with pre-existing diabetes are also much more stringent than those for non-pregnant adults with diabetes [1]. As such, the experiences and supports that women with pre-existing diabetes during pregnancy need likely differ from those with gestational diabetes and non-pregnant adults with diabetes. The objectives are threefold, as we integrated the quantitative and qualitative data within the overall mixed methods design: (1) to determine the predictors of glycemic control during pregnancy; (2) to understand the experience and diabetes self-management support needs during pregnancy among women with pre-existing diabetes; (3) to assess how self-management and support experiences helpe to explain glycemic control among women with pre-existing diabetes in pregnancy.

## Methods

This paper represents the mixed methods integration of a four-phased mixed methods sequential comparative case study [13, 14]. This is a complex mixed methods design that involves the integration of diverse types of data (quantitative and qualitative) to develop enhanced analyses and case descriptions of the topic of interest [13, 14]. This design provides detailed and contextualized data that is beneficial when there is a need to portray and understand complex variation regarding the subject under study [13]. Both the quantitative and qualitative phases received ethics approval from the Hamilton Integrated Research Ethics Board (REB #14–222 and #13,847).

### Quantiative phase

The quantitative phase consisted of an analysis of quantitative data collected as part of the ‘Assessing the Determinants of Pregestational Diabetes in Pregnancy: A Prospective Cohort Study.’ This study took place at the Maternal-Fetal Medicine clinic at McMaster University Medical Center in Ontario, Canada between April 2014 to November 2019. Consecutive convenience sampling was employed to recruit eligible participants who met the following criteria: (1) a diagnosis of type 1 or type 2 diabetes; (2) attending the Maternal-Fetal Medicine clinic at McMaster University Medical Centre clinic for obstetrical care; and (3) age 18 years or older. A total of 111 participants were recruited (type 1 diabetes, n = 55; type 2 diabetes, n = 56). Data were collected three times during pregnancy, between 0 and 16 weeks (time point 1 (T1)); 17–28 weeks (time point 2 (T2)); and 29–40 weeks (time point 3 (T3)). Participants completed a demographic questionnaire, surveys to measure self-efficacy for Diabetes scale), self-care behaviors (Summary of Diabetes Self-Care Activities and Measures) and satisfaction with medical care (Patient Assessment of Care for Chronic Conditions scale). Glycemic control was assessed via self-report of A1C and confirmation with medical charts. Descriptive statistics were completed to understand the distribution of participant demographic and clinical characteristics, and participant levels of self-efficacy, self-care and care satisfaction. Independent Samples t-Tests, Chi-squared tests and Fisher’s exact tests explored differences in baseline variable distribution, stratified by diabetes type. Linear mixed-effects modelling was used to explore trends in glycaemic control and examine self-efficacy, self-care and care satisfaction as predictors of A1C. Linear mixed-effects modeling was employed given non-independence in the data across timepoints – data was collected from the same participants at each timepoint. To control for potential confounding factors on the relationship between self-efficacy, self-care, care satisfaction and glycaemic control, we adjusted for participant age, diabetes duration, ethnicity, education level, household income and insurance coverage [15]. SPSS (IBM Corp. Released 2021. IBM SPSS Statistics for Macintosh, Version 28) was used to perform all statistical analyses.

### Qualitative phase

The qualitative phase was conducted between March and July 2022 and employed a qualitative description design. We used the principles of purposeful sampling to recruitment women aged 18 years or older, with type 1 and type 2 diabetes, who were currently or who were previously pregnant. A total of 12 women were recruited (type 1 diabetes, n = 6; type 2 diabetes, n = 6). The sample of women interviewed in the qualitative study attended the Maternal-Fetal Medicine clinic at McMaster University Medical Centre. However, they were not the same women included in the quantitative phase due to the different times of study conduct (April 2014 to November 2019 for the quantitative phase and March to July 2022 for the qualitative phase). Women participated in individual semi-structured interviews to describe their experience of managing diabetes and determine their needs regarding diabetes self-management education and support during pregnancy. Individual interviews were the primary means of data collection. The interviews were conducted face to face via videoconferencing (Zoom) with an approximate duration of 30–60 min. All interviews were audiorecorded. Baseline demographic and clinical characteristics were collected before the interview and supplementary field notes were written immediately after. The recorded audio was transcribed verbatim and imported into NVivo (NVivo. QSR International; 2020) for analysis. Conventional content analyses, as described by Hsieh and Shannon was employed [16].

### Mixed methods phase

In the context of this study, the use of a mixed-methods sequential comparative case study was ideal as we aimed to develop detailed and particularized information about the self-management experiences and preferences of women with pre-existing diabetes during pregnancy. Furthermore, we expected that the self-management experiences and preferences during pregnancy might vary based on diabetes type. Thus, the use of the mixed methods sequential comparative case study enabled us to understand the potential variation between these two populations. Our goal was to portray realistic and practical information about the evidence on this topic to guide subsequent research in designing, evaluating, and implementing self-management education and support interventions for this population.

We have previously published the study protocol [14], and the quantitative [15] and qualitative phases [16]. These provide details regarding our methodology. Briefly, the sequence of the mixed methods study was as follows: (1) Phase I: Prospective cohort; (2) Phase II: Planning the qualitative data collection; (3) Phase III: Qualitative descriptive; (4) Phase IV: Integration of quantitative and qualitative findings and case construction (Appendix A).

#### Mixed methods integration

The purpose of the mixed methods procedures was to integrate the quantitative and qualitative data. The goal was to develop a rich analysis and description of the diabetes self-management and support experiences and preferences during pregnancy of women with pre-existing diabetes and how these factors may help explain glycemia. Through the integration of the quantitative and qualitative data, cases were developed and refined based on these experiences and preferences. We used Creswell and Plano Clark’s recommendations for mixed methods research integration procedures to guide the mixing process [13]. Integration first occurred following the completion of the quantitative study when we analyzed the results to plan the interview guide. It also informed the participant selection approach for the qualitative study. The second integration, reported in the current paper, illustrates the sequential mixing of the quantitative and qualitative results and the development of cases to represent the main findings.

We incorporated Stake’s approach to instrumental and collective case studies [17, 18], which is utilized when the goal of the study is to facilitate an understanding of a phenomenon of interest [17], particularly for social sciences and human services research [19]. Stake’s approach allows for researcher flexibility and values the emergence of cases as the study progresses, aligning with the sequential ordering of our mixed methods approach [19]. We endeavoured to understand how factors related to self-management support (e.g., diabetes management behaviours and self-efficacy) and the pregnancy experience, help to explain glycemic control among women with pre-existing diabetes. In the instrumental and collective derivatives of Stake’s approach to case study research, cases are developed through categorical aggregation, based on repeated patterns and categories that emerge following researcher immersion in the data [18]. Also in keeping with Stake’s approach, we utilized methodological, data source and investigator triangulation to promote the validity of the case developemnt, as Stake’s approach values intituion and impression over rigid, preplanned case definitions and binding [18]. Finally, cases were defined through: (1) holistic (considers the connectivity of the phenomoenon and its context); (2) empirical (observations/data); and (3) interpretive (intuition or researchers) approaches, recognizing that each case is a complex and integrated entity, which is not limited or bound to working parts [17, 18]. Following our quantitative study, we developed an interview guide for the qualitative study to ensure that similar questions were asked across participants. Data source triangulation was achieved when participants provided similar answers across questions [18]. Investigator triangulation was attained when the first author conferred with the senior author throughout case construction [18]. To promote study transparency, quality and rigor, we followed the Good Reporting of a Mixed Methods Study tool (Appendix B) [20].

## Results

Our mixed-methods sequential comparative case study findings are presented in two approaches.

First, results are presented using a narrative approach, weaving together the quantitative statistics and the qualitative themes. Second, we created a joint display to present the quantitative results and qualitative findings alongside cases that were derived from the mixed methods integration.

### Quantitative results

In phase I (cohort), we explored the trends in glycemia and assessed self-efficacy [21], self-care [22] and care satisfaction [23] during pregnancy in 111 women (55, type 1 diabetes; 56, type 2 diabetes), across three time points during the perinatal period (time point one, zero to 16 weeks; time point two, 17 to 28 weeks; time point three, 29 to 40 weeks). Overall, the cohort’s average A1C was “at target” (≤ 6.5%) by time point two and remained “at target” at time point three. Measurements of self-efficacy and care satisfaction were relatively high among the cohort (Table 1). A one unit increase in self-efficacy (e.g., total score of 8 to a total score of 9) was associated with a mean reduction in A1C of 0.22% (95% CI -0.42, -0.02, *p* = 0.03). In using the self-care [21] tool, every one unit increase in the exercise sub-score (e.g., total score of four to a total score of five) was associated with a mean reduction in A1C of 0.11% (95% CI, -0.22, -0.01, *p*= 0.04). These associations were present after adjustment for confounders [15].

**Table 1:** Trends in A1C and results of questionnaires assessing diabetes self-efficacy, self-care and care satisfaction across time points, stratified by type of diabetes

	Total (***n*** = 111)	Type 1 Diabetes (***n*** = 55)	Type 2 Diabetes (***n*** = 56)
A1C, %			
T1	7.49 (7.23–7.77)	7.49 (7.15–7.83)	7.53 (7.11–7.95)
T2	6.41 (6.15–6.67)	6.72 (6.39–7.06)	6.08 (5.68–6.48)
T3	6.36 (6.11–6.60)	6.60 (6.28–6.93)	6.12 (5.78–6.49)
SED Scale			
T1	7.83 ± 1.31	8.06 ± 1.22	7.56 ± 1.38
T2	7.76 ± 1.33	7.85 ± 1.33	7.66 ± 1.34
T3	7.96 ± 1.20	8.12 ± 1.14	7.81 ± 1.26
SDSCA Scale			
T1	4.89 ± 1.56	4.95 ± 1.63	4.84 ± 1.49
Diet, general	3.80 ± 1.47	3.99 ± 1.42	3.59 ± 1.51
Diet, specific	2.92 ± 1.98	2.88 ± 1.96	2.97 ± 2.02
Exercise	6.42 ± 1.03	6.68 ± 0.70	6.12 ± 1.26
Glucose monitoring	2.56 ± 2.07	2.79 ± 2.26	2.29 ± 1.88
Foot care			
T2	4.96 ± 1.38	4.98 ± 1.44	4.85 ± 1.32
Diet, general	3.88 ± 1.18	4.02 ± 1.09	3.72 ± 1.26
Diet, specific	2.72 ± 1.75	2.62 ± 1.79	2.83 ± 1.73
Exercise	6.32 ± 1.10	6.54 ± 0.78	6.09 ± 1.34
Glucose monitoring	2.72 ± 2.05	2.76 ± 2.28	2.69 ± 1.79
Foot care			
T3	4.84 ± 1.41	4.72 ± 1.58	4.95 ± 1.23
Diet, general	3.84 ± 1.13	4.08 ± 1.16	3.59 ± 1.06
Diet, specific	2.92 ± 1.98	2.94 ± 1.86	2.89 ± 2.09
Exercise	6.50 ± 0.89	6.68 ± 0.64	6.33 ± 1.06
Glucose monitoring	2.99 ± 2.21	3.27 ± 2.32	2.71 ± 2.07
PACIC Scale			
T1	3.32 ± 0.88	3.48 ± 0.74	3.14 ± 1.00
T2	3.42 ± 0.80	3.29 ± 0.69	3.57 ± 0.89
T3	3.39 ± 0.81	3.26 ± 0.79	3.51 ± 0.82

Data are mean (95% CI) or mean ± SD; SED, self-efficacy for diabetes; SDSCA, summary of diabetes self-care activities; T1, time point one, zero to 16 weeks gestation; T2, time point two, 17 to 28 weeks gestation; T3, time point three, 29 to 40 weeks; PACIC, the patient assessment of chronic illness care

### Qualitative results

In phase III (qualitative description), we described the experience of managing diabetes during pregnancy and identified the self-management education and support preferences among 12 women (6, type 1 diabetes; 6, type 2 diabetes). We identified eight qualitative themes within two overarching categories: (1) themes describing patient experiences of managing diabetes in pregnancy; and (2) themes identifying preferences for diabetes self-management education and support during pregnancy. In general, women described the experience of managing diabetes during pregnancy as terrifying, isolating, mentally exhausting and they had feelings about being out of control. Preferences were expressed for individualized healthcare, mental health support and support from peers and the healthcare team [16].

### Mixed methods integration

**Fig. 1:**
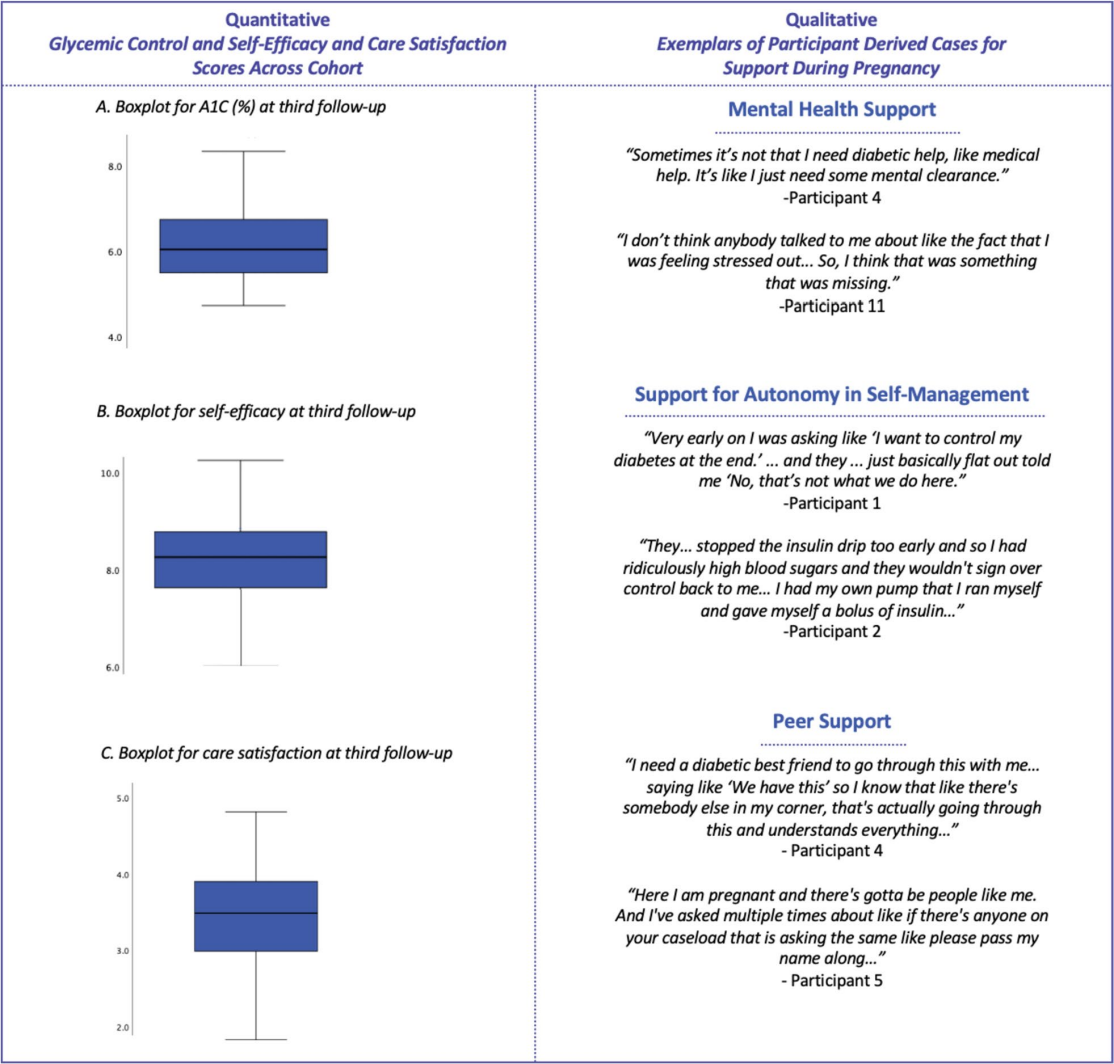
Joint-Display Table with Box Plots Depicting Quantitative Results (Good Glycemic Control, High Self-Efficacy, Low Satisfaction with Care) Side-by-Side with Qualitative Text of Participant-Derived Cases for Support during Pregnancy. Note: The box plots visually show the distribution of glycemic control (mean 6.36 [95% CI 6.11–6.60]), self-efficacy (range of the scale: 0 to 10; cohort mean 7.96 ± 1.20) and care satisfaction (range of the scale score: 0 to 5; cohort mean 3.39 ± 0.81) by the third follow-up

When conceiving this study, we examined the literature and hypothesized that glycemic control would be sub-optimal among this cohort. Thus, we planned to quantify glycemic control through the cohort study, use findings from the qualitative study to explain the reasons behind sub-optimal control and develop suggestions to optimize glycemia based on participant experiences and preferences. However, after analyzing and integrating the quantitative and qualitative data, we found that a different story emerged. In contrast to the hypothesized findings, the study cohort demonstrated high self-efficacy and achieved on target A1C. Thus, in addition to using the qualitative study to explore the reasons behind glycemic control, we explored the pregnancy experience through participant-derived suggestions and preferences for support. As the findings did not mirror the hypotheses, and in accordance with Stake’s case study approach [19], we modified how we constructed the cases from the plan outlined in the protocol [14]. Rather than developing cases based on variation in glycemia and covariates (e.g., self-efficacy, care satisfaction) across diabetes type, we used Stake’s approach that facilitated the highlighting of repeated patterns in the data to aid in the construction of cases for support in pregnancy that were participant-derived. Our mixed-methods apporach produced confirmatory and expansionary insights and as a result, three cases regarding participant-suggested self-management support preferences emerged: (1) Mental Health Support; (2) Support for Autonomy in Self-Management; and (3) Peer Support. Figure 1 depicts the constructed cases and supporting quantitative and qualitative data in a joint display. Below we have used a narrative approach that weaves together the quantitative statistics and the qualitative themes to further display the data integration.

Women in the study cohort demonstrated optimal glycemia across both types of diabetes, meeting the recommended national A1C guidelines of ≤6.5% [14]. By the third follow-up, the overall mean A1C remained “at target” at 6.36% (95% CI 6.11%, 6.60%). Confidence in self-management, represented by self-efficacy, was also relatively high and improved throughout pregnancy. By the third follow-up, the overall mean score on the self-efficacy scale was 7.96 (SD 1.20), out of a total possible score of 10 [15].

Upon reflecting on the qualitative findings in light of the quantitative results, we hypothesized that lower glycemic control may have manifested from women feeling out of control and fearing diabetes-induced pregnancy complications *(“You feel guilty when your blood sugar is high… you’re like, what important body part is being formed right now? And am I ruining it?”*). Unfortunately, the price of such tight control was an isolating and mentally exhausting pregnancy experience. *(“It’s like I can’t, I feel like I can’t be a normal person because I’m constantly having to check my blood sugar, constantly having to remember to take my insulin or if I’m going out like I have to make sure ‘OK do you have your insulin? Do you have your meter just in case your sensor goes wrong?’… I just I wish I was a normal person, but I’m not.”).* Such experiences contributed to the first constructed case, the desire for Mental Health Support (“*The piece that wasn’t a part of the high-risk clinic was the mental health piece and… I don’t think that people look at it so seriously. And I don’t think anybody talked to me about like the fact that like I was feeling stressed out… So, I think that was something that was missing”).* Possibly due to feeling out of control and scared, women put a lot of effort and research into their diabetes management, resulting in feelings of self-confidence *(“… My A1C was 5.3… you get to feel annoyingly confident because you’re like ‘Look at me, I’m doing better than a real pancreas’”*).

After women had worked hard to have tight control during pregnancy and were confident in their self-management, they wanted to be able to manage insulin administration during labour and delivery (*“Very early on I was asking like ‘I want to control my diabetes at the end”*; *“I had, you know built up the courage ‘cause you’re like, ‘I’m gonna tell this doctor how I want things done’ and so I was like ‘I want to be in charge. I don’t want to take off my pump, like under no circumstances.’”*). However, they found that they lacked support from the healthcare team to have autonomy in self-management during labour (“*They were like ‘That’s not what we do here. The protocol here is you will be put on an insulin IV’… Just basically flat out told me ‘No that’s not what we do here.’ …If I pushed back, it was always like ‘We have to do this or your baby’s at risk of dying…”*; *“So like at the end of the day I did end up having to go on the insulin IV… They did a terrible job. My blood sugar level was perfect when I went in… of course, my blood sugar went up. And it did not go down for the rest of labour and delivery…”*). These reported experiences were the basis for the development of the second case, the desire for Support for Autonomy in Self-Management.

Perceiving a lack of support from the healthcare team, women found that they needed to turn to their diabetes community for advice regarding diabetes management and thus the third constructed case was the desire for Peer Support (*“I have gastroparesis too… I found that my Endo and OB, that wasn’t even something that they’d ever really considered.”* ; *“I remember just asking like ‘All I want from you [endocrinologist] is like a suggestion on where my insulin like starting point should be… And he … wouldn’t help me at all… … I reached out to my community, to kind of get a read on what I thought was pretty standard [pump settings for labour and delivery]…”*). In addition to looking to the diabetes community for advice on diabetes management during pregnancy, women expressed a desire for regular companionship with fellow mothers with pre-existing diabetes who would understand their unique day-to-day struggles (*“My best friend is great. She’s wonderful …I can go to her and I can be like, ‘Oh my, my blood sugars are all over the place and everything’ but all she’s gonna say is ‘Oh, you know, like you got this, you can do it’… but I need a diabetic best friend… so I know that like there’s somebody else in my corner, that’s actually going through this and understands everything…”* ; *“It would have been nice to have that in-person connection to someone else like me… to connect to somebody else who would have been, like you know, someone who’s not just on Facebook. That would have been nice…”).*

## Discussion

The quantitative results indicate that participants were confident in diabetes management. As a result, they achieved optimal glycemia during pregnancy. This finding contrasted with expectations developed from the existing literature showing that women with type 1 and type 2 diabetes have difficulty reaching glycemic targets during pregnancy [24–26]. The qualitative findings, in contrast, show a different perspective: women appeared to be in significant mental distress and wanted support during pregnancy from peers and professionals. The finding of the need for peer support among those with diabetes in pregnancy has also been found in existing literature [27, 28]. Thus, integrating both findings resulted in an apparent discordance: women may be confident in diabetes self-management, yet, they still desired support during a challenging pregnancy. There was also an expressed desire for autonomy in diabetes management during labour and delivery. The quantitative results show that women maintained optimal glycemia during pregnancy by closely measuring carbohydrate intake and carefully administering insulin, changing the timing and dosing as they progressed through the pregnancy trimesters. However, once they began to labour, they were forced to surrender diabetes management to the healthcare team. The stripping of autonomy at this critical time was another contributor to the significant distress that women revealed. The findings of this integration have important implications for future research and policies that affect clinical practice focused on this population.

### Implications for clinical practice

Women in our study demonstrated high self-efficacy and achieved optimal glycemia. Their motivations were clear; the main priority was to avoid potential diabetes-induced complications for their infant. The fear of consequences for their infant and the strain of stringent self-management resulted in poor mental health. This is an example of the previously noted discordance: women appeared confident in diabetes management and achieved optimal glycemic control but they still had a strong desire for mental health support. Unfortunately, participants expressed that the healthcare team did not address their mental health concerns. Diabetes is often concomitant with mental health disorders among non-pregnant adults. Depression, for example, is two to three times more common among those with diabetes than those without diabetes [29]. Anxiety disorders are also more common among adults with diabetes, with a 20% higher prevalence when compared to the background population [30]. Pregnancy is a time when women are particularly susceptible to mental illness [31]. Research on mental health disorders and pre-existing diabetes in pregnancy is limited. However, a recent meta-analysis that studied women with gestational and pre-existing diabetes in combination found that they are at a significantly higher risk of developing depression during pregnancy than women without diabetes [31]. Few studies have focused on mental health among women with diabetes [31]. Thus, there is an opportunity for future research to examine more closely the prevalence of mental health disorders in pregnancy among women with pre-existing diabetes and to develop interventions to optimize mental health during this vulnerable time.

### Implications for research

Although the women were confident in their diabetes management and met their target glucose levels during pregnancy, they voiced a strong desire for peer support. The previously noted discordance remained: women were high functioning from a diabetes self-management perspective but still wanted connection and support. Some women explained that they engaged with peers in national and international online support groups for expectant mothers with pre-existing diabetes. Others described interacting with expectant mothers in their friend group who did not have diabetes. Neither of these social connections provided them with sufficient support. Thus, they wished for in-person peer support facilitated by the healthcare team. Research on peer support during pregnancy without diabetes is limited but shows that peer support may be beneficial for improving mental health outcomes. A narrative review of six studies on peer support and the development of postpartum depression showed some evidence for lower Edinburgh Postpartum Depression Scale scores following peer support interventions and reports of positive experiences and maternal satisfaction [32]. Other research indicated the beneficial effects of peer support on mood and anxiety may occur by decreasing feelings of isolation and stress [33]. To our knowledge, research focused on in-person peer support during pregnancy for pre-existing diabetes is limited to one study that assessed the need for peer support among those with gestational and type 2 diabetes, finding that almost half of the participants were interested in such an intervention [34]. Independent of diabetes, the literature indicates that up to 21% of women experience mood disorders during pregnancy and early parenthood [32]. Mental health disorders are even more common during a pregnancy complicated by pre-existing diabetes [30]. As such, the evidence indicates that peer support may contribute to reduced isolation and stress during pregnancy and early parenthood for women without diabetes [32], future research should focus on developing such interventions for those with pre-existing diabetes who are even more vulnerable to such feelings.

### Implications for policy

The quantitative results showed that the women in our study were confident in diabetes self-management, meeting recommended glycemic targets throughout pregnancy. However, once they began to labour, they were forced to allow the healthcare team to take over diabetes management. Women reported this was to the detriment of their physical and mental health: their glucose levels were allowed to run dangerously high, causing them significant stress and frustration. Thus, there was a strong desire for autonomy and diabetes self-management during labour. Currently, the policies at the regional centre where we conducted our studies do not support diabetes self-management during labour. Thus, the women in our study reported that they had to turn off their insulin pump, for example, and receive insulin via an intravenous controlled by the healthcare team. Management by the healthcare team was problematic because women revealed that when their glucose levels became too high, the team would not consider their requests to increase the insulin dose. This resulted in glucose levels near diabetic ketoacidosis, delayed hospital discharge and caused women to have to administer their insulin from home without the knowledge of the healthcare team. In other clinical settings, diabetes self-management using insulin pumps, for example, during short surgeries, is commonplace [35]. Established protocols exist in parts of the United States, Canada, Australia and Europe [36–39] and current research indicates that insulin pump use during labour is safe and may result in improved glycemic control [40–45]. Thus, it is imperative for policymakers and healthcare team members to use knowledge translation strategies that bridge the gap between evidence and clinical practice. Knowledge translation strategies that are effective for policymakers include providing information packages, one-on-one meetings and tailored summaries [46]. Effective strategies for healthcare team members include education workshops, webinars and in-services [46]. The implementation of these strategies to facilitate change in clinical practice is critical to allow for autonomy and diabetes self-management during labour.

### Strengths and limitations

Our mixed methods study has several strengths. First, through the quantitative phase, we demonstrated the prevalence and correlates of self-management support and glycemic control during pregnancy in women with type 1 and type 2 diabetes, offering insights related to women with type 2 diabetes: a population of women with limited research. We also revealed the diabetes self-management support experiences and preferences during pregnancy in this population in the qualitative phase. Finally, our mixed methods integration generated cases that provided deeper understandings regarding participant-derived support preferences related to diabetes self-management among women with type 1 and type 2 diabetes in pregnancy using methodological, data source and investigator triangulation to promote the validity of the case study.

However, our study also had limitations. First, the quantitative phase had several limitations, including the reliance on self-reported data surveys. Further details can be found in the published paper [15]. The qualitative phase also had limitations, including a relatively small sample size. This resulted in limited transferability of the study findings. Finally, the mixed methods integration had limitations. One of these was the need to find a method for case construction that better fit with the repeated and emerging findings in the data. In the original protocol, we described our plan to construct cases based on variation in glycemia and covariates (e.g., self-efficacy, care satisfaction) across diabetes type, using the Diverse Case Method [47] for case Sect [14]. This was based on the idea that glycemic control might be sub-optimal among our population and there could be variation in glycemic control and self-efficacy across the different diabetes types. As the study results revealed that this was generally not the case, we had to modify how we developed the cases. However, the guiding literature on mixed methods case study designs suggests a sequential and flexible approach to case development and emphasizes the importance of allowing the emergence of cases as the research progresses [13]. Thus, we used Stake’s approach to case construction that allowed for the development of cases based on emerging, repeated patterns in the data.

## Conclusions

In conclusion, overall, women with pre-existing diabetes in this cohort study are able to achieve tight glycemic control during pregnancy. They are motivated and display high self-efficacy in diabetes self-management. However, the achievement of optimal glycemia appeared to be driven by fear, which took a toll on their mental health and pregnancy experience. Thus, women desired mental health support, support for autonomy in self-management and peer support. We plan to use the findings from this study to provide the basis for the development, evaluation and implementation of interventions related to these participant-described support preferences.

### Supplementary Information

**Additional file 1: Appendix A. Figure S1.** Study Flow Diagram. **Appendix B. Table S1.** Application of the Good Reporting of a Mixed Methods Study (GRAMMS) Checklist^16^.

**Additional file 2: Table S1. **Participant Baseline Characteristics, Stratified by Type of Diabetes

**Additional file 3: Table S2. **Predictors of A1C, Stratified by Type of Diabetes.

## Data Availability

Study participants were advised that their raw data would remain confidential and not be shared publicly, particularly due to the sensitive nature of the interview questions. Upon reasonable request, the data are available from the corresponding author.
