# Supplementary material for: Supporting self-management in women with pre-existing diabetes in pregnancy: a mixed-methods sequential comparative case study
Source: BMC Nurs. 2024 Jan 2;23:1. doi: 10.1186/s12912-023-01659-1 (PMC10759746; doi:10.1186/s12912-023-01659-1)
Supplement: Supplementary file 1 — Additional file 1: Appendix A. Figure S1. Study Flow Diagram. Appendix B. Table S1. Application of the Good Reporting of a Mixed Methods Study (GRAMMS) Checklist16. [file 12912_2023_1659_MOESM1_ESM.docx]

**Appendix A**

**FIGURE S1. Study Flow Diagram**

**
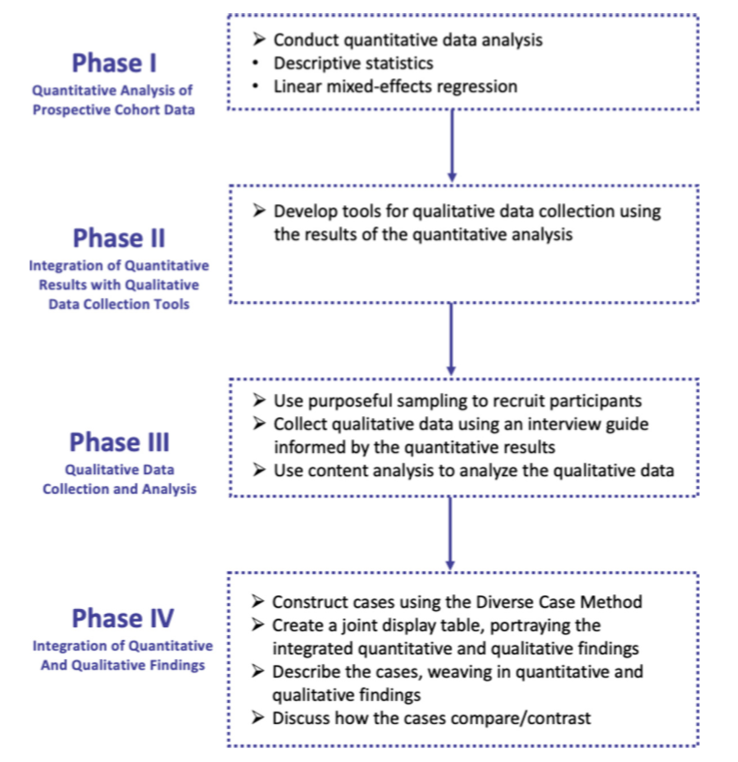
**

**Appendix B**

| **TABLE S1. Application of the Good Reporting of a Mixed Methods Study (GRAMMS) Checklist^16^** | |
| --- | --- |
| **GRAMMS Criteria** | **Study Information** |
| Describe Justification  for Using  Mixed Methods Approach | There is a need to utilize quantitative and qualitative methods to understand the complexities of diabetes self-management and support experiences and needs in pregnancy and link these to glycemic control among women with type 1 and type 2 diabetes. |
| Describe Study Purpose | Our objective was to explore how self-management and support experiences help explain glycemic control among women with type 1 and type 2 diabetes in pregnancy. |
| Describe Study Priority | The quantitative and qualitative phases had equal priority because both sets of data were compared to develop contextualized cases of participant-derived diabetes self-management support needs. |
| Describe Study Sequence | A sequential comparative case study design was used, wherein initial quantitative data was collected and analyzed, followed by collection and analysis of qualitative data. Both sets of data were then integrated to develop contextualized cases. |
| Describe Sampling, Data Collection,  and Analysis | Refer to the protocol for information regarding sampling, data collection, and analysis.^13^ |
| Describe the Integration | Refer to the protocol and the current paper for details regarding integration processes.^13^ |
| Describe Limitations of One Phase Caused by Presence of the Other | N/A |
| Describe Insights Gained  from Integration | The use of a mixed methods design enhanced the study results so that they are more than the sum of the quantitative and qualitative studies alone.  The association between self-management behaviours and glycemic control was determined and the impact that managing diabetes during pregnancy had on self-management behaviours and glycemic control was also explored. The resulting information lays the groundwork to guide subsequent research in designing, evaluating, and implementing self-management education and support interventions for this population in the future. |
