# Supplementary material for: Supporting self-management in women with pre-existing diabetes in pregnancy: a mixed-methods sequential comparative case study
Source: BMC Nurs. 2024 Jan 2;23:1. doi: 10.1186/s12912-023-01659-1 (PMC10759746; doi:10.1186/s12912-023-01659-1)
Supplement: Supplementary file 2 — Additional file 2: Table S1. Participant Baseline Characteristics, Stratified by Type of Diabetes [file 12912_2023_1659_MOESM2_ESM.docx]

| **TABLE S1. Participant Baseline Characteristics, Stratified by Type of Diabetes** | | | | |
| --- | --- | --- | --- | --- |
|  | **Total**  **(*N* = 111)*** | **T1D**  **(*n* = 55)** | **T2D**  **(*n* = 56)** | ***P*** |
| Age (years) | 30.23 (4.97) | 29.45 (4.69) | 30.98(5.15) | 0.105 |
| Ethnicity  African  East Asian  European  Hispanic  Middle Eastern  South Asian  Indigenous  Unsure  Other | 2 [1.9]  2 [1.9]  87 [81.3]  1 [0.9]  2 [1.9]  4 [3.7]  6 [5.6]  2 [1.9]  3 [2.8] | 2 [3.8]  1 [1.9]  47 [88.7]  0 [0]  1 [1.9]  0 [0]  1 [1.9]  1 [1.9]  1 [1.9] | 0 [0]  1 [1.9]  40 [74.1]  1 [1.9]  1 [1.9]  4 [7.4]  5 [9.3]  1 [1.9]  2 [3.7] | 0.243  1.000  0.053  1.000  1.000  0.118  0.205  1.000  1.000 |
| Marital Status  Single  Supportive Partner  Married | 12 [10.9]  26 [23.6]  72 [65.5] | 5 [9.1]  14 [25.5]  36 [65.5] | 7 [12.7]  12 [21.8]  36 [65.5] | 0.784 |
| Education Level  Grade School  High School  College/Trade  University  Other | 3 [2.7]  24 [21.6]  51 [45.9]  32 [28.8]  1 [0.9] | 0 [0]  13 [23.6]  19 [34.5]  22 [40.0]  1 [1.8] | 3 [5.4]  11 [19.6]  32 [57.1]  10 [17.9]  0 [0] | 0.243  0.609  0.017^†^  0.010^†^  0.495 |
| Household Income (dollars)  <20,000  20-40,000  41-60,000  61-80,000  81-100,000  >100,000 | 9 [8.3]  26 [24.1]  11 [10.2]  19 [17.6]  18 [16.7]  25 [23.1] | 4 [7.5]  9 [17.0]  7 [13.2]  10 [18.9]  10 [18.9]  13 [24.5] | 5 [9.1]  17 [30.9]  4 [7.3]  9 [16.4]  8 [14.5]  12 [21.8] | 0.606 |
| Employment  Not Working  Casual/Part-Time  Full-Time  Other | 30 [27.0]  24 [21.6]  55 [49.5]  1 [1.8] | 12 [21.8]  13 [23.6]  29 [52.7]  1 [1.8] | 18 [32.1]  11 [19.6]  26 [46.4]  1 [1.8] | 0.677 |
| Primiparous | 48 [43.2] | 25 [45.5] | 23 [41.1] | 0.641 |
| Singleton Gestation | 109 [98.2] | 54 [98.2] | 55 [98.2] | 1.000 |
| Used ART | 9 [8.1] | 1 [1.8] | 8 [14.3] | 0.032^†^ |
| Previous GDM |  |  | 7 [12.5] |  |
| Diabetes Duration (years) | 10.09 (8.61) | 15.13 (8.38) | 5.14 (5.39) | <0.000^†^ |
| Diabetes Treatment Method  Diet/Exercise  Oral Medications  Insulin Injections  Insulin Pump | 1 [0.9]  6 [5.4]  77 [69.4]  27 [24.3] | 0 [0]  0 [0]  28 [50.9]  27 [49.1] | 1 [1.8]  6 [10.7]  49 [87.5]  0 [0] | 1.000  0.027^†^  <0.001^†^  <0.001^†^ |
| SMBG at least Four Times per Day | 61 [57.0] | 29 [54.7] | 32 [59.3] | 0.635 |
| Insurance Coverage for Diabetes Supplies  ADP  Third Party  Other  None | 27 [25.0]  43 [39.8]  22 [20.4]  16 [14.8] | 25 [47.2]  15 [28.3]  10 [18.9]  3 [5.7] | 2 [3.6]  28 [50.9]  12 [21.8]  13 [23.6] | <0.001^†^  0.016^†^  0.704  0.009^†^ |

ADP, assistive devices program; ART, assisted reproductive technology; GDM, gestational diabetes mellitus; SMBG, self-monitoring of blood glucose; T1, time point 1; T2, time point 2; T3, time point 3; T1D, type 1 diabetes; T2D, type 2 diabetes.

Note: Mean (SD), Number [%]

*All totals do not equal 111 due to missing observations

†*p* value statistically significant at < 0.05
