# Supplementary material for: Supporting self-management in women with pre-existing diabetes in pregnancy: a mixed-methods sequential comparative case study
Source: BMC Nurs. 2024 Jan 2;23:1. doi: 10.1186/s12912-023-01659-1 (PMC10759746; doi:10.1186/s12912-023-01659-1)
Supplement: Supplementary file 3 — Additional file 3: Table S2. Predictors of A1C, Stratified by Type of Diabetes. [file 12912_2023_1659_MOESM3_ESM.docx]

**TABLE S2. Predictors of A1C, Stratified by Type of Diabetes**

|  | **Total**  **(*N* = 111)** | | **T1D**  **(*n* = 55)** | | **T2D**  **(*n* = 56)** | |
| --- | --- | --- | --- | --- | --- | --- |
|  | Unadjusted | Adjusted†,  ***P*** | Unadjusted | Adjusted†,  ***P*** | Unadjusted | Adjusted†,  ***P*** |
| SED  Scale | -0.18*  (-0.31, -0.05) | -0.06  (-0.19, 0.07),  0.339 | -0.16  (-0.34, 0.03) | 0.01  (-0.19, 0.19),  0.989 | -0.22*  (-0.40, -0.04) | -0.22*  (-0.42, -0.02),  0.034 |
| SDSCA  Scale  Diet, General  Diet, Specific  Exercise  SMBG  Foot Care | -0.19*  (-0.31, -0.08)  0.09  (-0.22, 0.03)  -0.07  (-0.16, 0.02)  -0.06  (-0.22, 0.10)  -0.02  (-0.09, 0.05) | -0.08  (-0.19, 0.03),  0.175  -0.05  (-0.17, 0.07),  0.414  -0.07  (-0.16, 0.01),  0.084  0.05  (-0.10, 0.20),  0.525  -0.03  (-0.10, 0.04),  0.422 | -0.18*  (-0.32, -0.04)  -0.11  (-0.23, 0.48)  -0.09  (-0.20, 0.02)  -0.21  (-0.52, 0.10)  -0.07  (-0.17, 0.02) | -0.11  (-0.24, 0.03),  0.111  -0.09  (-0.25, 0.08),  0.306  -0.11*  (-0.22, -0.01),  0.037  -0.07  (-0.40, 0.27),  0.692  -0.08  (-0.17, 0.01),  0.08 | -0.21*  (-0.39, -0.03)  -0.11  (-0.29, 0.07)  -0.04  (-0.18, 0.09)  -0.05  (-0.25, 0.15)  0.04  (-0.09, 0.16) | -0.04  (-0.03, 0.15),  0.667  -0.05  (-0.22, 0.12),  0.579  -0.06  (-0.19, 0.08),  0.431  0.09  (-0.08, 0.28),  0.282  0.06  (-0.05, 0.18),  0.283 |
| PACCC Scale | -0.08  (-0.28, 0.11) | -0.07  (-0.25, 0.12),  0.482 | 0.20  (-0.09, 0.50) | 0.07  (-0.22, 0.36),  0.611 | -0.17  (-0.44, 0.10) | -0.19  (-0.45, 0.07),  0.155 |
| Age  (years) | -0.07**  (-0.11, -0.04) | -0.03  (0.06, 0.01),  0.168 | -0.07*  (-0.12, -0.02) | -0.02  (-0.07, 0.04),  0.585 | -0.07*  (-0.13, -0.01) | 0.04  (-0.03, 0.10),  0.262 |
| Diabetes Duration (years) | -0.02  (-0.03, 0.02) | 0.01  (-0.01, 0.04),  0.305 | -0.02  (-0.05, 0.02) | -0.01  (-0.04, 0.04),  0.874 | -0.03  (-0.09, 0.02) | -0.02  (-0.06, 0.02),  0.380 |
| Ethnicity  *European*  *African*  *East Asian*  *Hispanic*  *Middle Eastern*  *South Asian*  *Indigenous*  *Unsure*  *Other* | Reference  -0.42 (-2.02,1.18)  2.27 (0.68, 3.87)*  0.13 (-1.87, 2.12)  1.61 (0.01, 3.21)*  -0.85 (-1.97, 0.27)  -0.59 (-1.63, 0.46)  0.79 (-0.62, 2.22)  -0.49 (-1.77, 0.79) | Reference  -0.51 (-2.24, 1.22),  0.561  3.19 (1.00, 5.39)*,  0.005  -2.80 (-4.97, -0.64)*,  0.012  1.47 (0.09, 2.85)*,  0.038  -0.75 (-1.69, 0.20),  0.122  -1.27 (-2.17, -0.36)*,  0.007  0.84 (-0.29, 1.96),  0.142  -0.52 (-1.59, 0.56),  0.342 | Reference  -0.62 (-2.21, 0.98)  0.79 (-1.32, 2.90)  --  0.44 (-1.68, 2.55)  --  --  -0.21 (-2.23, 1.80)  0.19 (-1.93, 2.29) | Reference  -0.74 (-2.48, 0.99),  0.392  --‡,  --  --‡,  --  --  0.36 (-1.22, 1.94),  0.641  -1.64 (-3.50, 0.22),  0.082 | Reference  --  4.18(1.79, 6.57)**  0.38 (-1.54, 2.29)  3.28 (0.89, 5.67)*  -0.55 (-1.66, 0.56)  -0.29 (-1.32, 0.73)  1.84 (-0.07, 3.76)  -0.64 (-2.20, 0.93) | Reference  --  --‡,  -2.64 (-0.04, 3.04),  0.055  -2.64 (-4.95, -0.34)*,  0.026  -0.17 (-1.47, 1.13),  0.796  0.20 (-0.84, 1.12),  0.693  1.50 (-0.04, 3.04),  0.055  -0.43 (-1.75, 0.88),  0.509 |
| Education Level  *Grade School*  *High School*  *College/Trade*  *University*  *Other* | Reference  -1.20 (-2.34, -0.07)*  -2.03 (-3.12, -0.93)**  -2.50 (-3.61, -1.39)**  -1.51 (-3.65, 0.63) | Reference  -2.30(-3.64, -0.97)**  <0.001  -2.81(-4.16, -1.46)**  <0.001  -3.36(-4.79, -1.93)**  <0.001  -2.48 (-4.69, -0.27)*,  0.028 | --  1.44 (0.87, 2.01)**  -2.25 (-3.38, -1.12)**  Reference  0.91 (-0.79, 2.59) | --  0.53 (-0.38, 1.45),  0.242  0.53 (-0.13, 1.19),  0.110  Reference  --‡, | Reference  -1.53 (-2.76, -0.30)*  -2.25 (-3.38, -1.12)**  -2.82 (-4.06, -1.59)**  -- | Reference  -3.63 (-5.09, -2.18)**,  <0.001  -3.90 (-5.37, -2.44)**,  <0.001  -4.39 (-6.00, -2.78)**,  <0.001  -- |
| Household Income (dollars)  *<20,000*  *20-40,000*  *41-60,000*  *61-80,000*  *81-100,000*  *>100,000* | Reference  0.09 (-0.69, 0.89)  -0.49 (-1.41, 0.42)  -0.25 (-1.08, 0.59)  -0.77 (-1.59, 0.06)  -0.84 (-1.64, -0.05)* | Reference  0.15 (-0.61, 0.92),  0.690  -0.09 (-0.98, 0.79),  0.835  0.02 (-0.82, 0.86),  0.975  -0.15 (-1.02, 0.72),  0.734  0.01 (-0.87, 0.88),  0.989 | Reference  0.20 (-0.85, 1.25)  -0.91 (-1.98, 0.17)  -0.85 (-1.88, 0.18)  -1.15 (-2.16, -0.13)*  -1.19 (-2.19, -0.21)* | Reference  0.39 (-0.69, 1.46),  0.471  -0.31 (-1.43, 0.82),  0.577  -0.07 (-1.15, 0.99),  0.885  -0.04 (-1.18, 1.11),  0.946  -0.09 (-1.29, 1.09),  0.872 | Reference  0.15 (-0.99, 1.29)  -0.39 (-1.90, 1.12)  0.23 (-1.05, 1.49)  -0.54 (-1.81, 0.73)  -0.63 (-1.82, 0.57) | Reference  0.67 (-0.37, 1.70),  0.195  0.40 (-1.07, 1.87),  0.581  0.69 (-0.51, 1.91),  0.249  0.27 (-1.08, 1.62),  0.681  0.38 (-0.88, 1.64),  0.543 |
| Insurance Coverage  *None*  *ADP*  *Third Party*  *Other* | Reference  -0.31 (-0.95, 0.33)  -0.31 (-0.91, 0.29)  0.52 (-0.16, 1.19) | Reference  0.18 (-0.49, 0.84),  0.599  0.09 (-0.48, 0.66),  0.758  0.76 (0.16, 1.36)*,  0.013 | Reference  -0.41 (-1.32, 0.50)  -0.33 (-1.27, 0.61)  1.19 (0.19, 2.19)* | Reference  -0.24 (-1.36, 0.88),  0.661  -0.25 (-1.34, 0.84),  0.642  1.34 (-0.19, 2.66),  0.087 | Reference  -1.06 (-2.84, 0.72)  -0.37 (-1.12, 0.39)  -0.21 (-1.13, 0.72) | Reference  -0.17 (-1.90, 1.56),  0.841  0.14 (-0.67, 0.96),  0.739  -0.40 (-1.25, 0.45),  0.344 |

ADP, assistive devices program; PACCC, Patient Assessment of Care for Chronic Conditions; SED, Self-Efficacy for Diabetes; SDSCA, Summary of Self-Care Activities; SMBG, self-monitoring of blood glucose; T1D, type 1 diabetes; T2D, type 2 diabetes. Note: Mean Change (95% Confidence Interval); **p* value statistically significant at < 0.05; ***p*-value statistically significant at <0.001; †Adjusted for participant age, diabetes duration, ethnicity, education level, household income and insurance coverage; ‡Only one participant, no adjusted value rep
